# Effects of I_Kur_ blocker MK-0448 on human right atrial action potentials from patients in sinus rhythm and in permanent atrial fibrillation

**DOI:** 10.3389/fphar.2014.00026

**Published:** 2014-03-04

**Authors:** Simone Loose, Judith Mueller, Erich Wettwer, Michael Knaut, John Ford, James Milnes, Ursula Ravens

**Affiliations:** ^1^Department of Pharmacology and Toxicology, Medical Faculty Carl Gustav Carus, Dresden University of TechnologyDresden, Germany; ^2^Clinic for Cardiac Surgery, Heart Center DresdenDresden, Germany; ^3^Xention Ltd.Cambridge, UK

**Keywords:** MK-0448, atrial fibrillation, Kv1.5, I_Kur_ blocker, human right atrial action potentials

## Abstract

Selective blockers of the Kv1.5 channel have been developed for the treatment of atrial fibrillation (AF), but little is known how these atrial-selective drugs affect human action potentials (APs). Therefore we have investigated the Kv1.5 blocker MK-0448 (*N*-{6-[(1S)-1-(4-fluorophenyl)-2,2-di(pyridin-3-yl)ethyl]pyridin-2-yl}methanesulfon- amide) in right atrial trabeculae from patients in sinus rhythm (SR), permanent AF (>6 months), and intermittent AF. MK-0448 blocked Kv1.5 current in an expression system and concentration-dependently elevated the plateau phase of atrial APs. In SR preparations stimulated at 1 Hz, MK-0448 (3 μM) shortened action potential duration at 90% of repolarization (APD_90_) and effective refractory period (ERP), but in permanent AF preparations, MK-0448 *prolonged* APD_90_ and ERP. The effects of MK-0448 in intermittent AF resembled those in SR preparations. Block of I_Ks_ is probably more prominent in AF because of reduced repolarization reserve due to AF-induced remodeling.

## Introduction

In search of safer antiarrhythmic drugs for the treatment of atrial fibrillation (AF), selective inhibitors of I_Kur_ have been designed as atrial-selective class III antiarrhythmic agents with therapeutic potential in AF. Since they target the Kv1.5 channels which are functional only in atria (Mays et al., 1995), these agents are considered to be devoid of proarrhythmic effects in ventricles and hence have received a lot of interest in the scientific community (for review see Ford and Milnes, 2008; Tamargo et al., 2009; Ravens, 2010; Ravens and Wettwer, 2011).

We have previously investigated the electrophysiological effects of several I_Kur_ inhibitors, including 4-aminopyridine, AVE0118, XEN-D0101, all of which elevated the plateau phase of human right atrial action potentials (APs) recorded in trabeculae of atrial appendage obtained from patients in sinus rhythm (SR) or AF (Wettwer et al., 2004; Christ et al., 2008; Ford et al., 2013). Interestingly, action potential duration (APD_90_) and effective refractory period (ERP) were shortened in SR but prolonged in AF preparations.

Recently, first-time-in-man results with another selective Kv1.5 inhibitor, MK-0448 were published (Pavri et al., 2012). MK-0448 failed to affect relative atrial ERP in healthy volunteers, and the authors concluded that I_Kur_ block cannot be expected to exert therapeutically useful antiarrhythmic effects in AF. Since nothing is known about the effects of MK-0448 in *ex-vivo* human atrial tissue, we investigated its effects on the shapes of atrial APs and ERP with standard microelectrode techniques in trabeculae isolated from right atrial appendages of patients in SR and AF, who had to undergo open heart surgery. In SR preparations, MK-0448 elevated the plateau potential and shortened APD and ERP at 1 Hz as expected from I_Kur_ blockers. We also confirm that MK-0448 effectively blocks Kv1.5 current in mouse fibroblasts stably expressing the *hKCNA5* gene.

## Methods

### Mouse fibroblasts

HK2BN9 cells expressing human Kv1.5 channels were kindly provided by Dr. Tamkun (Snyders et al., 1993). The cells were cultured in Dulbecco's modified eagle medium containing 10% fetal calf serum and 1% penicillin/streptomycin under atmospheric conditions of 5% CO_2_ at 37°C. 250 μg/ml G418 was added to the medium for selection. Expression of Kv1.5 was induced by treating the cells with 1 μM dexamethasone 24 h before an experiment. The cells were studied with a standard single electrode whole-cell voltage-clamp set-up as described previously (Radicke et al., 2006). The bath solution contained (in mM): NaCl 150, KCl 5.4, MgCl_2_ 2, CaCl_2_ 2.0, HEPES 10, glucose 10, pH 7.4, temperature 25°C. The electrode solution contained (in mM): KCl 40, potassium aspartate 80, NaCl 8, CaCl_2_ 2, MgATP 5, EGTA 5, GTP 0.1, HEPES 10, pH 7.4 adjusted with KOH. Series resistances were between 5 and 10 MΩ and were compensated by up to 85%. Cell capacitance was measured with small hyperpolarizing clamp steps from −40 mV to −42 mV, average value was 20.8 ± 1.2 pA/pF (*n* = 33) and did not differ between cells tested at 0.5 and 3 Hz. Voltage clamp pulse generation, data collection and analysis were performed with the ISO2 software (MFK, Niedernhausen, Germany). Data were not corrected for the junction potential which was 11.7 mV, as calculated with the JPCalc software (P.H. Barry, Sydney, Australia).

### Patients' diagnoses and medications

Atrial tissue was obtained from patients receiving cardiac surgery because of coronary artery or valve disease. All patients gave written informed consent. The study was approved by the ethic committee of the Medical Faculty of Dresden University of Technology (No. EK790799). The patients are listed in Table 1. The study included 13 drug-exposed and 9 TMC preparations from patients in sinus rhythm (SR group), 9 drug-exposed and 9 TMC preparations from patients with permanent atrial fibrillation (AF group, defined as permanent atrial fibrillation for ≥6 months at time of tissue collection), and 3 drug-exposed preparations from patients with intermittent AF (episodes of spontaneously terminating AF lasting for a few seconds up to several hours). The medication of the patients typically included ACE-inhibitors, β -blockers, nitrates, lipid-lowering drugs, and diuretics.

**Table 1:** **Characteristics of patients whose trabeculae were used for drug exposure**.

	**SR**	**Permanent AF**	**Intermittent AF**
*n*	22	17	5
male/female	19/3	12/5	5/0
Age, y	64.9 ± 2.0	71.5 ± 1.8	74.4 ± 1.6
BMI (kg m^−2^)	29.1 ± 1.1	30.1 ± 0.9	26.3 ± 0.9
Valve replacement, *n*	3	7	3
Bypass, *n*	17	6	1
Bypass and valve, *n*	2	4	1
Hypertension, *n*	19	16	4
Diabetes, *n*	6	9	0
Hyperlipidemia, *n*	9	11	2
Nicotin, *n*	5	1	0
CAD, *n*	19	11	2
LVEF, %	55.0 ± 2.9	47.5 ± 3.0	59.2 ± 4.5
***MEDICATION, n***			
Digitalis	0	3	2
ACE-inhibitors	15	10	4
AT1-blockers	4	3	0
β-Blockers	19	17	3
Ca^2+^ channel blockers	2	2	2
Diuretics	8	8	2
Nitrates	4	0	0
Lipid-lowering drugs	15	13	4
Antiarrhythmic drugs	1	0	1

*BMI, body mass index; CAD, coronary artery disease; LVEF, left ventricular ejection fraction; ACE, angiotensin-converting enzyme; AT1, angiotensin receptor-1 blocker*.

### Experimental set-up

Small pieces (50–100 mg in weight) of human right atrial appendages were transported to the laboratory in a special Ca^2+^-free transport solution at 20–25°C, composition in mM: 100 NaCl, 10 KCl, 1.2 KH_2_PO_4_, 5 MgSO_4_, 50 taurine, 5 MOPS, 30 2,3-butane-dione monoxime (BDM), pH 7.0. Either free-running trabeculae (<1 mm in diameter and 2–5 mm in length) or trabeculae together with attached atrial wall were dissected and mounted on the bottom of a 5 ml organ bath perfused with 50 ml of recirculating, oxygenized Tyrode's solution at a flow rate of 7 ml/min at 36 ± 1°C (composition in mM: 126.7 NaCl, 0.42 NaH_2_PO_4_, 22 NaHCO_3_, 5.4 KCl, 1.8 CaCl_2_, 1.5 MgCl_2_, pH 7.4 when equilibrated with 5% CO_2_ in O_2_). Preparations were electrically stimulated at a basal rate of 1 Hz with isolated square-wave stimuli of 1 ms duration, 2 times threshold intensity. Transmembrane potentials were recorded with glass microelectrodes filled with 2.5 M KCl. Tip resistances of the electrodes were between 20 and 80 MΩ. Both timing of the driving stimuli and pre-processing of the transmembrane potential responses were carried out with a computer-aided action potential recording system (Wettwer et al., 2004). Chart for Windows, Version 7.3.5, recording programme (ADInstruments Pty Ltd., Castle Hill, Australia) was used for analysis of action potential parameters.

Each experiment was preceded by a 60 min equilibration period during which the preparations were allowed to stabilize and residual BDM was completely washed out. Numerical values of the main action potential parameters had to remain constant, i.e., within a range of ±5% for at least 10 min. When the microelectrode moved out of the cell, a new impalement was made at the same place and the experiment was continued if the new recording did not differ from the old one by visual judgment and showed less than 3% difference upon final numerical analysis.

### Measurement of effective refractory period

Effective refractory period (ERP) was measured by a single extra stimulus interpolated after trains of 10 regular stimulation pulses at the basal frequency of 1 Hz with decreasing intervals in steps of 5 ms from the last regular pulse until the extra stimulus failed to elicit an action potential. Following a short recovery period the preparation was exposed to MK-0448 (3 μM) at 1 Hz for another 60 min and ERP was tested once more in the presence of drug.

### Stock solutions and drug application

MK-0448 was synthesized by Xention Ltd. Cambridge UK. In a few experiments, MK-0448 was applied in cumulatively increasing concentrations by directly adding aliquots from a 10 mM stock solution with DMSO as solvent to 50 ml of the circulating bath medium. The DMSO concentration never exceeded 0.2%, a concentration which did not induce any significant change when applied alone. Preparations were exposed to each concentration of MK-0448 for 20 min.

### Analysis of action potential parameters

The following standard parameters of atrial APs were analyzed: resting membrane potential, RMP (mV), action potential amplitude, APA (mV), action potential duration at 20, 50, and 90% of repolarization, APD_20_, APD_50_ and APD_90_ (ms), maximum rate of depolarization, *dV*/*dt*_max_ (V/s) and the “plateau potential” defined as the mean absolute membrane potential (mV) in the time window between 20 and 30% of APD_90_ (PLT_20_). Preparations with resting potentials less negative than −65 mV and amplitudes below 80 mV were discarded.

### Statistical analysis

GraphPad Prism (version 4.03) was used for statistical analysis. In the concentration-response curves (see Table 2) the values obtained at increasing concentrations were compared to pre-drug controls by One-Way ANOVA followed by Bonferroni's multiple comparison test. The individual action potential parameters of SR and permanent AF preparations in the presence of 3 μM MK-0448 were compared to pre-drug controls by paired Student's *t*-test (see Table 3). Differences are considered statistically significant for *P* < 0.05.

**Table 2:** **Concentration-dependent effects of MK-0448 at 1, 3, and 10μM (1 Hz)**.

**SR**	**Control**	**1.0μM**	**3.0μM**	**10.0μM**	**Wash**
	***n* = 5**	***n* = 5**	***n* = 5**	***n* = 5**	***n* = 4**
APD_90_ (ms)	307.6±10.2	269.7±8.2	266.9±12.2	260.9±19.5	271.6±13.7
APD_50_ (ms)	116.3±28.6	136.5±33.1	149.6±35.6	167.4±20.9^*^	154.6±17.4
APD_20_ (ms)	5.4±1.5	51.3±20.5	92.0±23.2	92.5±23.8^**^	63.7±25.3
PLT_20_ (mV)	−20.4±3.4	−4.0±6.2^***^	+2.8±6.9^***^	+3.4±6.8^***^	−6.0±6.7
APA (mV)	95.3±2.7	94.5±2.3	93.2±3.6	88.6±4.7	86.5±7.2
RMP (mV)	−74.2±1.9	−74.5±4.0	−72.8±3.5	−70.9±4.6	−70.0±5.3
*dV*/*dt*_max_ (V/s)	300.8±24.4	267.3±29.8	269.2±27.9	191.8±58.3	186.3±54.3
**Permanent AF**	**Control**	**1.0μM**	**3.0μM**	**10.0μM**	**Wash**
	***n* = 5**	***n* = 5**	***n* = 5**	***n* = 5**	***n* = 5**
APD_90_ (ms)	190.8±11.8	200.7±9.3	215.2±11.1	221.4±15.2^*^	222.9±17.0
APD_50_ (ms)	101.6±7.4	125.7±7.5	143.1±8.4^*^	147.8±11.1^**^	145.9±11.8
APD_20_ (ms)	31.8±8.8	55.7±4.0	73.4±5.8^**^	78.2±7.1^**^	71.7±8.4
PLT_20_ (mV)	−9.9±2.4	3.0±1.8^*^	9.5±2.2^***^	11.6±4.3^***^	4.7±4.2
APA (mV)	100.9±3.6	104.0±2.2	101.4±3.7	98.2±6.7	96.9±5.4
RMP (mV)	−79.2±3.3	−78.9±2.6	−75.7±3.0	−70.9±3.9^***^	−74.6±2.4
*dV*/*dt*_max_ (V/s)	211.0±36.4	204.0±40.9	177.8±23.6	147.3±32.2	200.1±37.2

*APD_90_, APP_50_, APD_20_, action potential duration at 90, 50, and 20% of repolarization, respectively; PLT_20_, average plateau potential at 20–30% of APD_90_; APA, action potential amplitude; RMP, resting membrane potential; dV/dt_max_. maximum rate of depolarization. Means ± s.e.m. from 5 experiments in which all concentrations indicated were tested*.

* P < 0.05,

** P < 0.01,

*** *P < 0.001; One-Way-ANOVA followed by Bonferroni's multiple comparison test (drug vs. pre-drug control)*.

**Table 3:** **Effects of MK-0448 (3μM) on action potential parameters and effective refractory period (ERP) of human right atrial trabeculae from patients in SR and AF (stimulation frequency 1 Hz)**.

**SR**			
**AP parameter**	**Control, *n* = 6**	**MK-0448, 3μM, *n* = 6**	**Wash, *n* = 4**
ERP (ms)	329.2±15.8	293.3±19.4^*^	276.3±20.5
APD_90_ (ms)	318.2±8.2	265.3±16.0^*^	251.8±20.3
APD_50_ (ms)	139.0±9.7	176.5±11.6	116.3±40.8
APD_20_ (ms)	4.0±0.6	53.2±22.0	25.1±22.0
PLT_20_ (mV)	−16.0±1.2	11.3±2.6^***^	−10.4±7.6
APA (mV)	98.7±1.6	96.8±2.4	95.3±3.6
RMP (mV)	−76.8±1.4	−75.7±1.1	−76.5±1.2
*dV*/*dt*_max_ (V/s)	250.2±26.9	263.8±39.7	283.0±31.5
**Permanent AF**			
**AP parameter**	**Control, *n* = 4**	**MK-0448, 3μM, *n* = 4**	**Wash, *n* = 4**
ERP (ms)	222.5±13.1	295.0±19.3^**^	297.5±34.1
APD_90_ (ms)	218.3±12.1	275.0±13.9^*^	271.0±16.5
APD_50_ (ms)	104.3±12.4	190.0±10.9^**^	185.0±14.1
APD_20_ (ms)	35.7±11.5	109.5±9.3^***^	108.0±16.9
PLT_20_ (mV)	−4.3±4.5	14.9±4.4^***^	1.1±11.6
APA (mV)	99.5±2.3	98.5±3.3	97.5±4.6
RMP (mV)	−79.5±0.5	−76.8±2.0	−77.5±2.5
dV/dt_max_(V/s)	237.5±58.1	261.3±95.1	282.3±88.8

*For abbreviations, see legend to Table 2*.

* P < 0.05,

** P < 0.01,

*** *P < 0.001; Paired Student's t-test (drug vs. pre-drug control)*.

## Results

### Effects of MK-0448 on Kv1.5 current

**Figure 1:**
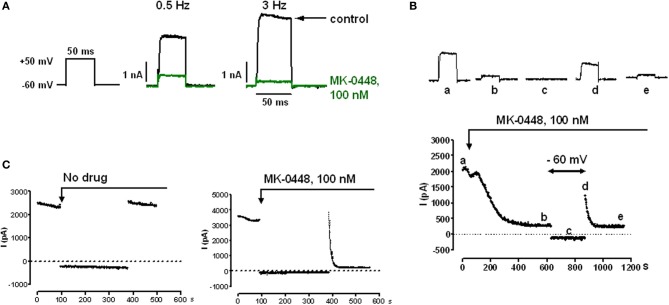
**Effects of MK-0448 on Kv1.5 currents measured in HK2BN9 cells at 25°C. (A)** Voltage clamp and current traces obtained before (black) and after superfusion of cells with 100 nM MK-0448 (green) for 10 min. Rate of clamp pulses as indicated. **(B)** Onset of blocking effect of MK-0448 (100 nM) on peak Kv1.5 current and recovery from block during 5 min at holding potential of −60 mV. Individual current traces at top were obtained at the time points indicated by small letters in the diary plot at the bottom. **(C)** Onset of drug effect following exposure during 5 min of continuous clamping at −60 mV. Step frequency: 3 Hz.

Kv1.5 currents, which was measured during step potential changes from a holding potential of −60 mV to various test potentials between −40 and +80 mV had a threshold potential of −30 mV, activated rapidly and hardly inactivated within 500 ms (data not shown). For drug testing we chose 50 ms duration voltage-clamp steps to +50 mV that elicited mean current amplitudes of 85.0 ± 10.5 pA/pF at 0.5 Hz (*n* = 16) and 110.6 ± 16.9 pA/pF at 3 Hz *n* = 17, *P* = 0.2133). MK-0448 (100 nM) blocked Kv1.5 current amplitude in a frequency-dependent manner (Figure 1A). At 0.5 Hz, the block reached a steady-state within 5–10 min of drug application and partially recovered during a 5-min period during which the cell was continuously clamped at −60 mV (Figure 1B). When cells were exposed to MK-0448 during a 5 min period held at −60 mV, to maintain channels in the closed-state, I_Kv1.5_ of the first post-rest pulse was not different from pre-rest. Subsequent pulses in the presence of drug revealed a rapid onset of inhibition, increasing with each pulse until reaching steady-state (Figure 1C). MK-0448 (100 nM) reduced I_Kv1.5_ from 82.3 ± 31.5 pA/pF to 22.4 ± 6.0 pA/pF (*n* = 5) within 3 min of continuous voltage-clamp steps at 0.5 Hz, and from 113.8 ± 52.7 pA/pF to 12.9 ± 2.5 pA/pF at 3 Hz (*n* = 4). These results are consistent with MK-0448 inhibition of Kv1.5 being contingent upon channel activation.

### Effects of MK-0448 on action potentials

**Figure 2:**
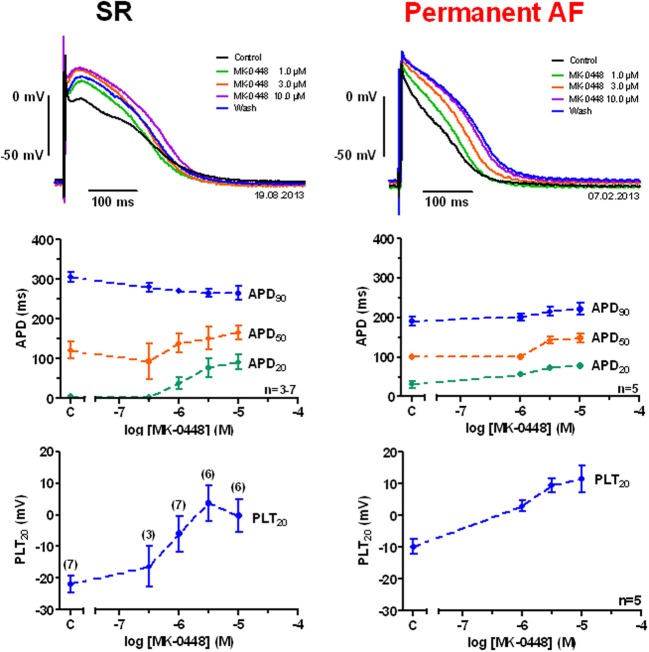
**Concentration-dependent effects of MK-0448 on action potentials recorded in right atrial trabeculae from patients in sinus rhythm (SR) and permanent atrial fibrillation (AF). Top:** Individual tracings for typical experiments, recorded in the presence of increasing concentrations of compound (see color code). Exposure time at each concentration: 20 min; control: pre-drug control. **Middle** and **bottom**: Mean values ± s.e.m. from 7 experiments in SR and 5 in AF, of action potential duration at 90, 50, and 20% of repolarization (APD_90_, APD_50_, and APD_20_) and plateau potential (PLT_20_). Frequency of stimulation: 1 Hz. Please note, that not all preparations were exposed to the full range of concentrations (numbers of preparations indicated in brackets).

Action potentials recorded in right atrial trabeculae from patients in SR had the typical spike-and-dome configuration (Figure 2), in permanent AF tissue the shape was triangular and is the result of electrical remodeling [for review see (Dobrev and Ravens, 2003)]. The most striking concentration-dependent effect of MK-0448 (1–10 μM) on SR action potentials at the basal stimulation frequency of 1 Hz was a robust elevation in plateau potential (PLT_20_) (Figure 2; Table 2). In this set of experiments, the APD_90_ tended to be shortened (not significant) whereas APD_20_ and APD_50_ were prolonged, reaching the level of statistical significance only at the highest concentration (see Table 2). Resting membrane potential (RMP), action potential amplitude (APA) and maximum depolarization velocity (*dV*/*dt*_max_) were not significantly affected by MK-0448 (see Table 2). Before and after drug treatment all preparations followed the stimulation, even at the highest concentration.

**Figure 3:**
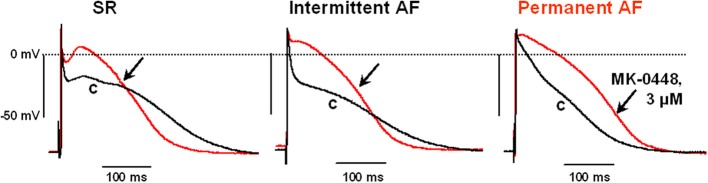
**Effects of MK-0448 (3 μM, red trace, arrow) on action potentials recorded in right atrial trabeculae from patients in sinus rhythm (SR), intermittent atrial fibrillation (iAF) and chronic atrial fibrillation (AF)**. Original recordings from one preparation in each group is shown. c, pre-drug control (black trace). Stimulation frequency 1 Hz.

The effects of MK-0448 on ERP were studied only at 3 μM. This concentration produced maximum changes within 40–50 min of drug application, and these were partially reversible after 60 min of washout. In 6 preparations from patients in SR, APD_90_, and ERP were significantly shortened (Figure 3; Table 3). Similar effects were observed in 2 preparations from patients in intermittent AF (Figure 3).

In permanent AF trabeculae, MK-0448 also significantly elevated the plateau potential to a similar extent as in SR, but in contrast to SR, *prolonged* APD at the three analyzed repolarization levels (Figures 2, 3; Table 3). In permanent AF preparations, APA, RMP, and *dV*/*dt*_max_ were also not significantly affected, with exception of RMP with 10 μM MK-0448.

Because of limited available we could only study the effects of MK-0448 in 2 trabeculae from patients in intermittent AF, a typical recording is shown in Figure 3; 3 others were used as TMCs. Similar to the results in SR preparations, MK-0448 shortened APD_90_ and ERP in the 2 intermittent AF preparations.

In time-matched control experiments with tissue from patients in SR, intermittent AF, and AF, action potential parameters did not change significantly (data not shown).

## Discussion

The characteristic changes in action potential parameters observed with MK-0448 in human right atrial trabeculae are consistent with block of Kv1.5 channels as reported previously for several drugs [e.g., 4-AP, (Wang et al., 1993; Amos et al., 1996); DPO-1, (Regan et al., 2006); AVE-0118, (Christ et al., 2008); XEN-D0101, (Ford et al., 2013)].

MK-0448 was described as highly selective for expressed Kv1.5 channels [IC_50_ ~10 nM; (Pavri et al., 2012)] over a wide variety of standard cardiac ion channels. hERG channels are about 10,000-fold, inward rectifier current Kir2.1 and Kir3.1/3.4 about 1000-fold, and Kv7.1 (I_Ks_) some 10- to 100-fold less sensitive than Kv1.5 (Pavri et al., 2012). The voltage-clamp data in a heterologous expression system confirm that MK-0448 (100 nM) produced a robust block of human Kv1.5 with a rather slow onset of action and partial recovery from block at −60 mV. Although our results do not allow precise characterization of drug-channel interaction they clearly indicate that resting channels recover from block and activation is necessary for development of block, suggesting preferential binding to open and/or inactivated channels.

In previous experiments, we demonstrated that low concentrations of 4-aminopyridine selectively inhibit I_so_ (I_Kur_) in human atrial myocytes (Wettwer et al., 2004). This blocker elevated the plateau potential in SR preparations (EC_50_~15 μM). In addition, 4-AP at concentrations that are considered to be selective for I_Kur_ block significantly shortened APD_90_ in SR but prolonged APD_90_ in permanent AF trabeculae (Wettwer et al., 2004). The most prominent effects of MK-0448 on human atrial APs, i.e., elevation of plateau potential, shortening of APD_90_ in SR and prolongation of APD_20_, APD_50_ and APD_90_ in permanent AF, are in line with our previous findings with other I_Kur_ inhibitors, so that we are confident to conclude that MK-0448 does indeed block I_Kur_ in human atrial trabeculae.

Unlike inhibition of hERG channels, inhibition of I_Ks_ was not associated with a proarrhythmic ventricular effect in animal models (Lynch et al., 1999). Nevertheless, modulation of I_Ks_ by MK-0448 could also reduce repolarization reserve in the ventricle and become arrhythmogenic in ventricles under appropriate conditions (Lynch et al., 1999). It is important to note that it is difficult to assess the ventricular proarrhythmic risk of a drug that modulates I_Ks_ in healthy volunteers with normal ventricular repolarization reserve. Therefore, the atrial selectivity of MK-0448 would need to be carefully assessed in patients.

The shortening of APD_90_ and ERP by Kv1.5 blockers in SR is proposed to be caused by the marked elevation of the plateau potential in to a voltage range where more hERG channels are activated, and, though inactivated rapidly, can recover from inactivation at more negative potentials and thus hasten final repolarization (Gintant, 2000).

Besides distinct alterations in shapes of action potentials, loss of rate-adaptation of ERP and APD_90_ to frequency has been recognized as a hallmark of atrial remodeling both in patients (e.g., Attuel et al., 1982; Daoud et al., 1996) and in *ex vivo* studies (Franz et al., 1997; Dobrev and Ravens, 2003). In our present study and in the recent “first-time-in-human” study with MK-0448 all electrophysiological testing was in a frequency range much below that of AF and one could argue that based on our SR data that it is not surprising that MK-0448 did not prolong AERP in healthy volunteers (Pavri et al., 2012).

Experimental and clinical evidence suggests that atrial fibrillation is due to micro-reentry triggered by ectopic foci located in the proximity of the pulmonary veins (Haissaguerre et al., 1998). Pharmacological interventions for interrupting micro-reentry have to prolong the effective refractory period, whilst suppression of ectopic foci requires reduction of excitability. Provided that MK-0448 does indeed selectively block I_Kur_, it is more likely to increase atrial refractoriness (in AF) than to reduce excitability, although it is not clear how the marked elevation of the plateau amplitude relates to the prolongation of ERP in AF. It must be emphasized, however, that the overall shape of the cardiac action potential is determined by multiple ion conductances, hence interpretation of action potential data cannot be conclusive with respect to which ion channels contribute to a particular change.

In SR, we even observed *shortening* of the ERP with MK-0448 and we cannot judge whether this is an anti- or proarrhythmic effect in the atria. Indeed this can only be answered in a prospective Phase 2 trial in AF patients. However, it is worth noting that no proarrhythmia was reported for MK-0448 (Pavri et al., 2012) or shortening of AERP in healthy volunteers. Other Kv1.5 inhibitors, e.g., XEN-D0101, that have been evaluated in the clinic have also not reported pro-arrhythmia (atrial or ventricular) in healthy volunteers (Ford et al., 2013). Genetic studies revealed an association of “loss-of-function” mutations in the *KCNA5* gene encoding for Kv1.5 with familial “lone” AF (Olson et al., 2006). However, in this study there was incomplete genotyping/phenotyping of family members and no evidence for primary linkage. Indeed, the likelihood of observing these data by chance alone is between 10 and 15%. A recent study of more than 300 patients with early-onset lone AF revealed 3 “gain-of-function” mutations in *KCNA5* in addition to several loss-of-function mutations, supporting the hypothesis that AF susceptibility may be enhanced by either change in KCNA5 function (Christophersen et al., 2013).

### Study limitations

In some human atrial preparations, APD does not remain stable during prolonged superfusion with saline solutions (typically, ~2 h).—The classification of patients into SR and AF may not always be reliable, i.e., the SR group may include atrial tissue from patients that have had unrecognized episodes of paroxysmal or recent onset AF.

## Conclusion

In summary, the I_Kur_ blocker MK-0448 consistently elevates the action potential plateau in human atrial trabeculae irrespective of the initial shapes of the control action potentials and whether the preparations were obtained from patients in SR or permanent AF. In SR preparations, MK-0448 produced shortening of APD_90_ and ERP, but in permanent AF tissue, prominent APD_90−_ and ERP-prolonging effects were observed with the drug. For prevention of persistent AF recurrence, prolongation of ERP by MK-0448 in AF preparations is certainly a highly desirable mode of action that may protect the atrial myocardium during the phase of reverse remodeling after cardioversion although if due to I_Ks_ modulation this may come at a risk of inducing ventricular proarrhythmia. It remains to be determined if selective I_Kur_ drugs (i) are more effective at prolonging ERP at higher atrial rates, (ii) pharmacologically convert recent onset AF, (iii) reducing AF burden in paroxysmal AF, and (iv) prevent the recurrence of persistent AF.

### Conflict of interest statement

James Milnes and John Ford are employees of Xention Ltd., John Ford is co-founder and holds stock in Xention Ltd., Ursula Ravens has acted as consultant to Xention. Simone Loose, Judith Mueller, Michael Knaut, Erich Wettwer have nothing to disclose.
